# Dataset on the relationship between consumer satisfaction, brand attitude, brand preference and purchase intentions of dairy product: The case of the Laayoune-Sakia El Hamra region in Morocco

**DOI:** 10.1016/j.dib.2020.106172

**Published:** 2020-08-13

**Authors:** Omar Boubker, Khadija Douayri

**Affiliations:** a Department of Management, Laayoune Higher School of Technology, Ibn Zohr University, Quartier 25 Mars BP 3007, Ibn Zohr University, Agadir, Morocco; b Higher School of Technology - Mohammed First University Oujda, Morocco

**Keywords:** Relationship marketing, Brand preference, Brand attitude, Consumer satisfaction, Purchase intention, PLS-SEM approach

## Abstract

This data article focuses on the relationship between consumer satisfaction, brand attitude, brand preference, and purchase intentions. The data was collected from dairy products consumers within the Laayoune-Sakia El Hamra region in Morocco. The research data are collected via an on a self-administered online questionnaire, from a sample of 195 Moroccan consumers of dairy products (Sakia brand). The data were analysed using a structural equation modeling method under the Partial Least Squares approach (PLS-SEM). Data analysis was performed using SmartPLS 3 software.

**Specifications Table**

**Table:** 

Subject	Marketing
Specific subject area	Relationship marketing:
Type of data	Tables and Figures
How data were acquired	A survey was carried out among Moroccan consumers of the dairy products offered by the Sakia agricultural dairy cooperative.
Data format	Raw, analyzed and descriptive data
Parameters for data collection	The sample consisted of consumers of the dairy products offered by the Sakia agricultural dairy cooperative (Sakia brand). The choice of this brand is justified by its strong cultural reputation among the population of the Laâyoune Sakia El Hamra region. The questionnaire was self-administered via the Google Forms tool during the month of April 2020.
Description of data collection	The survey link was disseminated via social networks (Facebook, WhatsApp).
Data source location	Laayoune City/ Laayoune Sakia El Hamra region Morocco; Latitude: 31.7945 Longitude: -7.0849.
Data accessibility	Repository name: Mendeley Data Data identification number: 10.17632 / nj9r2k8zp9.4 Direct URL to data: https://data.mendeley.com/datasets/nj9r2k8zp9/4

**Value of the Data**

• The dataset is useful because it helps to explain the effect of consumer satisfaction on brand attitude, brand preference and purchase intentions in the context of dairy industry.
• This dataset can be used to enlighten dairy industry brand managers on the importance of consumer satisfaction as a key factor to improve brand attitude and brand preference.
• This dataset provides insights into diverse aspects of consumer satisfaction, brand attitude, brand preference, and purchase intentions.
• With this data, academics and students will find a practical example of the SEM-PLS approach use in the relationship marketing area.
• The dataset can be adapted for use in other similar contexts such as soft drinks industry.

## 1 Data Description

The constructs and measurement items used in this data article were drawn from previous relationship marketing research (Table 1). A questionnaire survey was carried out among Moroccan consumers of the dairy products offered by the Sakia agricultural dairy cooperative (Sakia brand). The questionnaire was self-administered via the Google Forms tool during the months of April 2020. The data raw and the research questionnaire are available in mendeley data on: https://data.mendeley.com/datasets/nj9r2k8zp9/4.

**Table 1:** Measurement instruments

Variables	Adapted Items		Scale
Consumer satisfaction [1]	*CS1*	I am satisfied with my decision to customize the product from this brand.	5-point Likert satisfaction scale
	*CS2*	Consuming dairy products from this brand is a good idea	
	*CS3*	I am happy that I customized the product from this brand.	
	*CS4*	Consuming dairy products of this brand is a good choice	
	*CS5*	I am disappointed with this brand (reverse scoring)	
Brand attitude [2]	*BA1*	Unpleasant/ pleasant	5 degree Osgood differential scale
	*BA2*	Bad/ good	
	*BA3*	Unfavourable/favourable	
Brand preference [3]	*BP1*	I like this brand better than any other brand of dairy products.	5-point Likert-scale
	*BP2*	I would consume this brand more than any other brand of dairy products...	
	*BP3*	I would be inclined to buy dairy products from this brand over rather than from other brands.	
	*BP4*	This is my preferred brand overall brands of dairy products	
Purchase intentions [4]	*PI1*	What is the likelihood that you would recommend this brand to someone close to you? (1) low; (5) high	5-Likert-type scale
	*PI2*	You were again consuming dairy products offered by this brand ?	
	*PI3*	Would you recommend dairy products offered by this brand to a friend and/or relative?	
	*PI4*	Were you buying products offered by this brand?	

The profile and characteristics of the SAKIA brand consumers who participated in this survey are illustrated in Table 2. A total of 195 responses from consumers were received, including 110 women (56.4%) and 85 men (43.6%). The age of the respondents varies between 16 and 60 with an average age of 24.9587. Finally, 78.6% of the respondents are single, 19.5% married and 2% divorced.

**Table 2:** Profile and characteristics of respondents (n = 195)

Attributes	Characteristic	Frequency	Percentage (%)
Gender	Male	85	43,60
	Female	110	56,40
Marital status	Single	153	78,50
	Divorced	4	2,00
	Married	38	19,50

## 2 Experimental Design, Materials and Methods

**Fig. 1:**
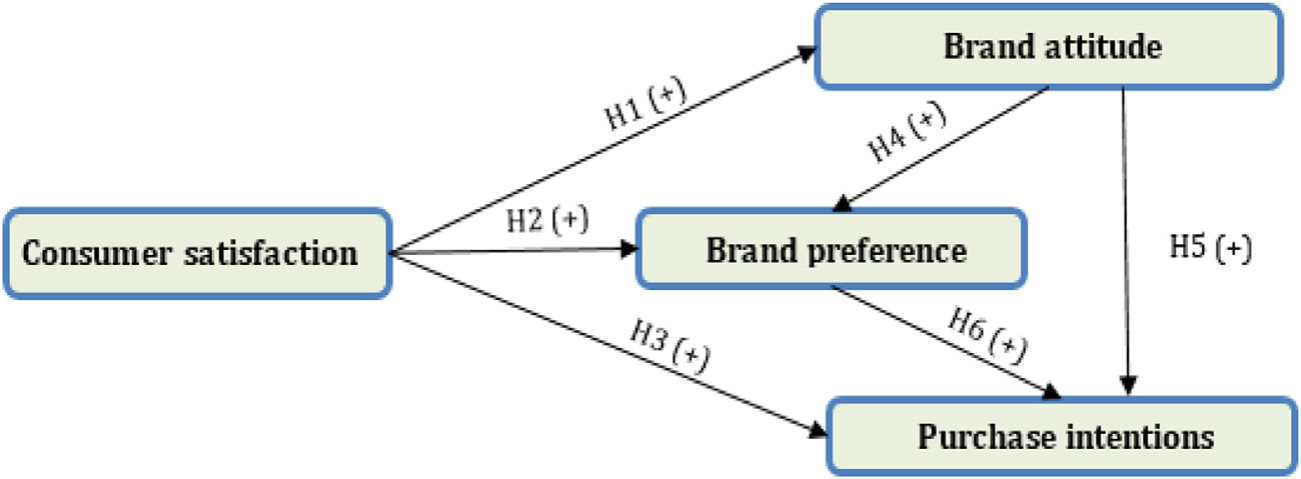
Conceptual framework.

Fig. 1, illustrates the research hypotheses based on previous marketing research. The developed model suggests that consumer satisfaction explains brand attitude (H1), brand preference (H2) and purchase intentions (H3). In addition, brand attitude contributes to the explanation of brand preference (H4) and purchase intentions (H5). Finally, the model shows a direct positive and significant relationship between brand preference and purchase intentions (H6). To test the hypotheses and the research model, we used the structural equations method under the Partial Least Squares approach (PLS-SEM). As indicated in Table 3, the implementation of this method takes place in two steps [5]. The first one consists of assessing the reliability and validity of the measurement models, while the second step focuses on assessing the fit of the structural model. Data analysis was performed using SmartPLS 3 software [6].

**Table 3:** Partial Least Squares Path Modeling Method

Steps	Criteria	Accepted value	
Step 1. Evaluation of the Measurement Models (Outer model)			

Convergent validity	Individual item reliability	Cronbach's α > 0.7	
	Composite reliability	CR > 0.7	
	Factor Loadings	Loadings > 0.7	
	Average Variance Extracted	AVE > 0.5	
Discriminant validity	Variable correlation (Root square of AVE)	The AVE of each latent construct should higher than the construct's highest squared correlation with any other latent construct.	
	Cross loadings	The loading of an indicator on its assigned latent variable should be higher than its loadings on all other latent variables	

Step 2. Evaluation of the Structural Model (Inner model)			

Coefficient of determination of the endogenous constructs (R-square)		R² < 0.19	Unacceptable
		0.19 ≤ R² < 0.33	Weak
		0.33 ≤ R² < 0.67	Moderate
		R² ≥ 0.67	Substantial
Effect size (f^2^)		f^2^ < 0.02	No effect size
		0.02 ≤ f^2^ < 0.15	Small effect size
		0.15 ≤ f^2^ < 0.35	Medium effect size
		f^2^ ≥ 0.35	Large effect size.
Predictive relevance (Q^2^)		Q^2^ > 0	Acceptable validity
		Q^2^ < 0	No validity
Hypotheses Testing (Path Coefficient & t-value)		t-value = 1.96 → Sig. at p-value < 0.05*; t = 2.58 → Sig. at p-value < 0.01**; t = 3.29 → Sign. at p-value < 0.001***.	
Goodness of Fit of the Model (GoF)$GoF=\sqrt{\left(\bar{R^{2}}\times \bar{AVE}\right)}$		GoF < 0.10	No fit
		0.1 ≤ GoF < 0.25	Small
		0.25 ≤ GoF < 0.36	Medium
		GoF ≥ 0.36	Large

**Fig. 2:**
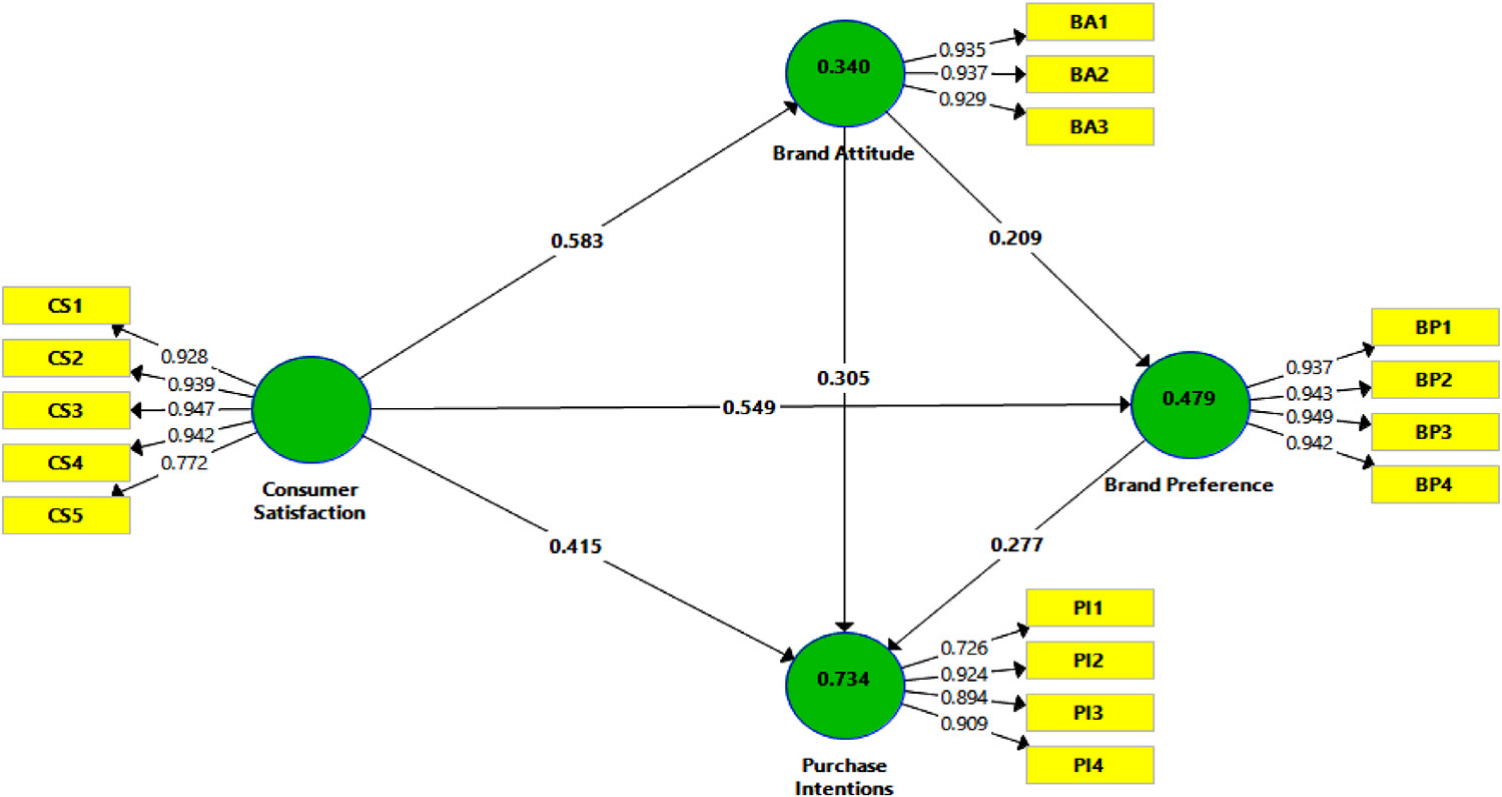
Measurement and structural model - Output SmartPLS.

Table 4 shows the summary of the convergent validity, according to several criteria: individual item reliability (Cronbach's alpha > 0.7), composite reliability (CR > 0.7), factor loadings (Outer loading > 0.7) and average variance extracted (AVE > 0.5). The outputs illustrated in the tables (Table 5 & Table 6) make it possible to verify the discriminant validity of the latent variables, in terms of the variable correlation [7] and the cross-loading criterion [8]. The SEM-PLS estimation for the measurement and structural model are shown in Fig. 2.

**Table 4:** Convergent validity

Constructs	Items	Outer loading (>0.7)	Cronbach's alpha (>0.7)	Rho-A (>0.7)	CR (>0.7)	AVE (>0.5)
Consumer satisfaction (CS)	CS1	0.928	0.945	0.953	0.959	0.825
	CS2	0.939				
	CS3	0.947				
	CS4	0.942				
	CS5	0.772				
Brand attitude (BA)	BA1	0.935	0.927	0.927	0.953	0.872
	BA2	0.937				
	BA3	0.929				
Brand Preference (BP)	BP1	0.937	0.958	0.959	0.970	0.889
	BP2	0.943				
	BP3	0.949				
	BP4	0.942				
Purchase Intentions (PI)	PI1	0.726	0.886	0.889	0.923	0.751
	PI2	0.924				
	PI3	0.894

**Table 5:** Discriminant validity (Fornell-Larcker criterion).

Constructs	BA	BP	CS	PI
Brand Attitude (BA)	0.934*			
Brand Preference (BP)	0.529	0.943*		
Consumer satisfaction (CS)	0.583	0.671	0.908*	
Purchase Intentions (PI)	0.694	0.717	0.779	0.867*

⁎ *Root square of AVE*

**Table 6:** Discriminant Validity - Loading and Cross-Loading criterion.

	Brand Attitude	Brand Preference	Consumer satisfaction	Purchase Intentions
BA1	0.935	0.490	0.540	0.636
BA2	0.937	0.495	0.540	0.644
BA3	0.929	0.497	0.553	0.663
BP1	0.497	0.937	0.647	0.690
BP2	0.474	0.943	0.631	0.657
BP3	0.531	0.949	0.633	0.706
BP4	0.491	0.942	0.619	0.649
CS1	0.597	0.608	0.928	0.760
CS2	0.466	0.642	0.939	0.704
CS3	0.574	0.658	0.947	0.722
CS4	0.545	0.638	0.942	0.748
CS5	0.451	0.486	0.772	0.586
PI1	0.818	0.512	0.555	0.726
PI2	0.578	0.693	0.749	0.924
PI3	0.472	0.634	0.652	0.894
PI4	0.527	0.634	0.726	0.909

The values of the coefficient of determination of the couple endogenous latent variables; brand attitude and brand preference are moderated, which are 0.340 and 0.479 respectively. The purchase intentions are explained at 73.4% (R² = 0.734 ≥ 0.67). This indicates a substantial level of determination. The size effect (f^2^) values are all acceptable (Table 7).

**Table 7:** Effect size.

Constructs		Effect size (f^2^)	Signification
Consumer satisfaction	Brand Attitude	0.515	Large effect size
	Brand Preference	0.382	Large effect size
	Purchase Intentions	0.308	Medium effect size
Brand Attitude	Brand Preference	0.055	Small effect size
	Purchase Intentions	0.219	Medium effect size
Brand Preference	Purchase Intentions	0.151	Medium effect size

**Fig. 3:**
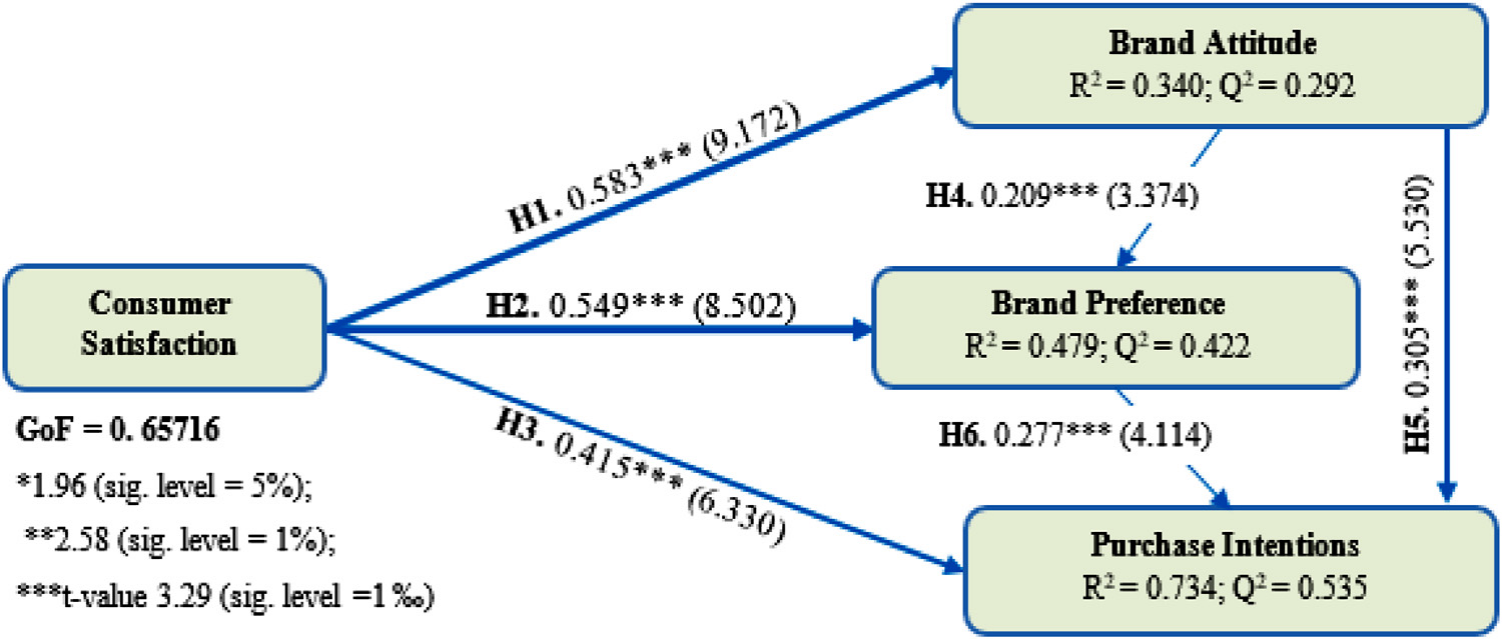
Structural equation model analysis.

The Predictive relevance (Q^2^) values are all greater than zero, which makes it possible to consider that the model has an acceptable predictive quality [5]. The fit of the global model (GoF) is very strong, with a value of 0.657 [9]. According to SmartPLS outputs, it turns out that consumer satisfaction greatly contributes to the explanation of brand attitude, brand preference and purchase intentions. Likewise, the brand attitude has a positive and significant effect on brand preference and purchase intentions (Fig. 3). On the other hand, brand preference has a significant effect on the purchase intentions.

## Ethics Statement

The consent of respondents was obtained. The online questionnaire was completely anonymous and does not contain any information allowing identifying the respondent.

## Declaration of Competing Interest

The authors declare that they have not known competing financial interests or personal relationships, which have, or could be perceived to have, influenced the work reported in this article.

## Appendix Supplementary materials

Image, application 1
